# Importance of Ventilation

**Published:** 1868-03

**Authors:** 


Importance of Ventilation.—Dr. J. Henry Bennet, in his lectures "On the Treatment of Pulmonary Consumption by Hygiene, Climate, and Medicine," published in the *Lancet*, mentions two remarkable incidents, demonstrating the importance of fresh air. He says:

"Some years ago I was riding in the Highlands of Scotland with a local proprietor, when we came upon a village of well-built stone houses with slate roofs, which strongly contrasted with the miserable shanties or hovels generally met with. On my complimenting him on his rebuilt village, he told me that he had acted for the best in erecting these good weather-proof houses for his tenants, but that, singular to relate, they had proved more unhealthy than the miserable dwellings which their occupants previously inhabited. Fever and other diseases had proved rife in the new houses. On examination, I found that the windows were fastened, and never opened; and I have no doubt that their comparative unhealthiness was in reality owing to their being quite weather-tight, and consequently unventilated. In the miserable hovels they previously inhabited, if the rain of heaven came in, so did pure air.

"The other fact is narrated by Prof. Hind, in a recent interesting work on Labrador. Consumption appears to be all but unknown to the natives living wild in the fastnesses of this desolate region, in tents made of spruce branches imperfectly lined with skins, and more or less open on all sides to the external air; although they are exposed to famine and every species of hardship. But when these same natives come down to the St. Lawrence to take a part in the fisheries, occupy well-built houses, and, being well paid, live in comparative luxury, most of them in the course of a year or two become consumptive and die miserably. I am fully impressed with the idea that the development of the disease under these circumstances is the result of their living in closed houses in a vitiated atmosphere, as it no doubt is in our own towns."

